# Supplementary material for: Analysis of PPARγ Signaling Activity in Psoriasis
Source: Int J Mol Sci. 2021 Aug 10;22(16):8603. doi: 10.3390/ijms22168603 (PMC8395241; doi:10.3390/ijms22168603)
Supplement: Supplementary file 1 [file ijms-22-08603-s001.zip › Supplemental materials_Analysis of PPARg signaling activity in psoriasis/Pathway models/Models images and html files/Model of PPARG signaling in psoriasis/96877.html]

beta-1,3-glucan


# Small Molecule beta-1,3-glucan

|  |  |
| --- | --- |
| URN | urn:agi-cas:9051-97-2 |
| Total Entities | 0 |
| Connectivity | 351 |
| Name | beta-1,3-glucan |

---

|  |  |
| --- | --- |
| Pathway | Dectin-1 (CLEC7A) Signaling |
|  | Antigen-Presenting Cell Role in Asthma |
|  | Model of PPARG signaling in psoriasis |
|  | 1\_Th2 cell response in asthma |

---

|  |  |
| --- | --- |
| MedScan ID | 1280131 |

---

|  |  |
| --- | --- |
| Alias | 9051-97-2 |
|  | dp540 |
|  | guardoran |
|  | drieline |
|  | polysaccharides laminarin |
|  | immustim |
|  | tak-n |
|  | sodium laminarin |
|  | beta-1,3-polyglucose |
|  | beta 1,3 d glucan |
|  | beta 1,3 dextro glucan |
|  | polysaccharide 13140 |
|  | beta 1,3 glucane |
|  | (1-3)-beta-d-glucan |
|  | glucan f |
|  | pc-3 type glucan |
|  | beta-1,3-glucan |
|  | 1,3-beta-glucan |
|  | vitastim-taito |
|  | glucan dp 540 |
|  | tak (polysaccharide) |

---

|  |  |
| --- | --- |
| CAS ID | 9051-97-2 |

---
